# *In Vitro* Anti-cancer Activity of Adipose-Derived Mesenchymal Stem Cells Increased after Infection with Oncolytic Reovirus

**DOI:** 10.34172/apb.2021.034

**Published:** 2020-04-20

**Authors:** Abouzar Babaei, Hossein Bannazadeh Baghi, Akram Nezhadi, Zahra Jamalpoor

**Affiliations:** ^1^Trauma Research Center, Aja University of Medical Sciences, Tehran, Iran.; ^2^Infectious and Tropical Diseases Research Center, Tabriz University of Medical Sciences, Tabriz, Iran.; ^3^Immunology Research Center, Tabriz University of Medical Sciences, Tabriz, Iran.; ^4^Neuroscience Research Center, Aja University of Medical Sciences, Tehran, Iran.

**Keywords:** Oncolytic virus, Reovirus type 3 Dearing, Mesenchymal stem cell, Glioblastoma cancer

## Abstract

***Purpose:*** Reovirus type 3 Dearing (ReoT3D), a wild type oncolytic virus (OV) from the *Reoviridae* family, kills KRAS mutant cancer cells. However, the use of OVs has faced with some limitations such as immune responses, and delivery of OVs to the tumor sites in systemic therapy. To solve this, and also to increase the anti-cancer effects of these OVs, mesenchymal stem cells (MSCs) might be used as an effective vehicle for OVs delivery. In this study, we examined the anti-cancer effects of human adipose derived-MSCs (AD-MSCs) as a vehicle of ReoT3D against human glioblastoma cells.

***Methods:*** Here, AD-MSCs were characterized and toxicity of ReoT3D on them was determined by 3-(4, 5-dimethylthiazol-2-yl)-2, 5-diphenyltetrazolium bromide (MTT) assay. Then, capability of AD-MSCs for virus production was assessed by real-time polymerase chain reaction (PCR), and different in vitro anti-cancer experiments were applied for our anti-cancer purposes.

***Results:*** Our results from toxicity assay revealed that the isolated and provoked AD-MSCs were resistant to nontoxic concentration multiplicity of infection (MOI) >1 pfu/cells of ReoT3D. In addition, the results indicated that AD-MSCs were susceptible for virus life cycle complementation and were capable for production of virus progenies. Furthermore, our results showed that AD-MSCs had oncolysis effects and increased the anti-cancer effects of ReoT3D.

***Conclusion:*** AD-MSCs as a susceptible host for oncolytic reovirus could increase the anti-cancer activity of this OV against glioblastoma multiforme (GBM) cell line.

## Introduction

Glioblastoma multiforme (GBM) is one of the most common and devastating cancers of brain and central nervous system (CNS).^1-3^ Surgery, radiotherapy and chemotherapy (such as temozolomide) are conventional approaches used for treatment of GMB. Unfortunately, statistics showed that the patients suffering from GMB only have 15 months median survival rate and GMB has still been considered as incurable disease.^4,5^ Therefore, finding of effective approaches for precise treatment of the disease is an urgent need.

Oncolytic viruses (OVs) due to having selectively targeting and replication in canner cells have been introduced as a new effective anti-cancer agent.^6^ Recently, the clinical therapeutic usage of OVs against cancer cells has gained a great attention.^7^ OVs may be natured or engineered with tropism for cancer cells that selectively replicate, which kill cells and spread in the tumor environment.^6,8^

Oncolytic reovirus is a wild type member of the *Reoviridae* family with double-stranded RNA genome virus, which usually does not cause severe disease in humans.^9^ Three strains of reovirus (Dearing, long, and Jones) have naturally anti-tumor activity; because of high oncolysis ability of Dearing (ReoT3D), most of the clinical trials have focused on this strain.^10,11^ It targets the cancer cells containing activated KRAS signaling pathway,^12^ a genetically aberrations that frequently were seen in the different human cancers with variable activation rate; it also replicate in these types of cancer cells.^13,14^ However, some factors adversely affect effectiveness of OVs in cancer therapy such as factors affecting systemic delivery of OVs, affecting intratumoral spread of OVs, and affecting anti-tumor immune responses.^8^ To get rid of these obstacles, some types of cells such as irradiated cell lines, immune cells, dendritic cells and mesenchymal stem cells (MSCs) have been proposed to use as a new delivery system for OVs.^15^

In recent years, scientists have paid a great attention to the vehicle usage of MSCs, which have inherent tumor tropism that migrates to tumor sites and targets tumor cells.^16^ MSCs are a subpopulation of stem cells that can be isolated from adipose, bone marrow, and skeletal muscle tissues.^17^ Passing capability through the vessels and inherent tropism to the tumor sites of MSCs introduced this type of stem cell as a best choice for delivery of OVs.^18-20^ The human adipose derived-MSCs (AD-MSCs), which have easily isolation from surgical wastes and large scale expanding in the cellular laboratories, have been mostly used in the cell vehicle studies.^21^

In this study, to enhance the capability of characterized AD-MSCs for ReoT3D delivery, the in vitro behavior and anti-cancer properties of AD-MSCs loaded with OV against the U-251 cell line were assessed. Moreover, the susceptibility of AD-MSCs as a host for production of reovirus progenies was evaluated.

## Materials and Methods

### Cell culture

The human *glioblastoma* U251 cell line and murine L929 cell line were cultured in high glucose Dulbecco’s modified Eagle medium (DMEM) (Sigma, USA) that supplemented with 10% of fetal bovine serum (FBS) (Gibco, USA), 1X of Non-Essential Amino Acids solution (NEAA) (Sigma, USA) and glutamine (Glu) (Sigma, USA) and 1% penicillin and streptomycin (pen-strep) (Sigma, USA). The cultured cells were passaged and assessed for presence of mycoplasma by polymerase chain reaction (PCR) assays, every 4 months, and the results verified no presence of the infection.

### Virus propagation and titration

Initially, L929 cells were infected, at 80% confluency, at a multiplicity of infection (MOI) 1 of ReoT3D (gifted by Dr Lee at the University of Alberta) that followed by incubation at 37 ˚C with 5% CO_2_. It was propagated as previously described.^22^ Then, supernatant of infected cultures was harvested after 48 hours by triplicate cycle of freeze-thawing; collected supernatants were purified and virus titrations determined by tissue culture infectious dose 50 (TCID_50_) according to the previous study.^23^

### AD-MSCs culturing

Primary AD-MSCs, at passage 0, were obtained from Stem Cell Technology Research Center, Tehran, Iran and cultured in DMEM/*F-12* media: 1:1 mixture of DMEDM and Ham’s Nutrient Mixture *F-12* with 10% FBS and 1% pen-strep at incubation atmosphere. After 24 hours incubation, the adherent cells at P0 routinely washed with PBS and supernatant of cultures were replaced with supplemented DMEM/F-12 media every 3 days until getting maximum confluences of AD MSCs. The phase-contrast microscopic images of cultures were taken every 24 hours. Finally, AD-MSCs cultures at passage 3 were used for characterization and further experiments.

### AD-MSCs characterization 

The isolated AD-MSCs were confirmed by flow cytometry assay. Briefly, passage 3 of MSCs at >80% confluency was harvested from the flasks. After washing with PBS, the cells were counted and their aliquots resuspended in fluorescence-activated cell sorting (FACS) buffer consisted of FBS (2%) and 0.1% NaN_3_ in PBS. The cells were incubated at 4°C for 20 minutes in the dark with fluorescent-conjugated mouse anti-human CD73 (*Cat No*: 11-0739-42), CD34 (Cat No: 11-4321-42), CD44 (*Cat No*: 17-0441-82), and CD90 (eBioscience, Germany, Cat No: 11-0909-42). Next, they were washed twice with FACS buffer, fixed with paraformaldehyde (1%, Cat, Sigma, No: 30525-89-4), and then the mentioned surface antibodies of AD-MSCs were quantified in a flow cytometry (FACSCalibur, BD Biosciences, San Jose) and raw data analysis was performed using FlowJo version 7.6 software.

Also, the AD-MSCs were differentiated toward adipogenic and osteogenic direction, which confirms the multipotentiality feature of these cells.^24^ For adipocyte differentiation, 2 ×10^3^ of AD-MSCs at passage 3 were cultured in the each well of six-well plate and then adipogenic differentiation medium (*Cat No*: A1007001) containing DMEM, FBS (10%), pen (100 U/mL) – strep (100 µg/mL), insulin (10 µg/mL), indomethacin (100µM), Dexamethasone (0.1µM), and 1-methyl-3-isobutylxanthine (IBMX) (0.5 mM) was added into the test wells. The cultures supernatant were replaced and cells were fed with fresh differentiation media containing indomethacin as an induction factor every 2 days until 8 days. Finally, the cells were fixed with paraformaldehyde (4%) and stained with Oil red (Behnogen, Tehran, Iran, *Cat No*: 102419) as described previously.^25^

For osteogenic differentiation, 2 ×10^3^ of AD-MSCs at passage 3 were placed in the each well of six-well plate and then fresh osteogenic differentiation medium (*Cat No*: A1007201) containing DMEM, FBS (10%), pen (100 U/ml) – strep (100 µg/mL), dexamethasone (0.1µM), B-Glycerol phosphate (10 mM), and Ascorbic acid 2-phosphate (0.2 mM) was added into the test wells. The cultures were fed with fresh differentiation media every 3 days during 21days and eventually the cells were stained by Alizarin Red (Behnogen, Tehran, Iran, *Cat No*: TMS-008-C) as described before.^25^

### AD-MSCs viability in ReoT3D infection

Cytotoxicity effect of ReoT3D on AD-MSCs was determined by 3-(4, 5-dimethylthiazol-2-yl)-2, 5-diphenyltetrazolium bromide (MTT) assay. To this end, 1 ×10^4^ of AD-MSCs at Passage 2 were seeded in each well of 96-well plate and incubated in supplemented DMEDM/F-12 medium at 37°C with 5% CO_2_. When test cells reached 80%-85% confluency, they were infected with ReoT3D at MOI: 0 to 20 pfu/cells, and incubated for 24 and 48 hours. Next, each of the test wells incubated for 2 hours with 50 ul of MTT (1 mg/mL stock concentration) (Sigma, USA, *Cat No*: 298-93-1). Then, the supernatant was removed from the wells and 50 ul of Dimethyl sulfoxide (DMSO) (Sigma, USA, *Cat No*: 506008) added into each well, and MTT assay was followed by incubation for 30 minutes. Finally optical density (OD) of samples was measured using spectrophotometer; the raw data were used for toxicity analysis.

### AD-MSCs capability to virus production by Real-time PCR assay

For real time PCR (qRT-PCR) assay, AD-MSCs (2 ×10^5^ cells/well) were seeded in six-well plate, they were infected with MOI: 1 pfu/cells of ReoT3D at >80% confluency and incubated for 2 hours. The supernatant of infected AD-MSCs containing the unabsorbed viruses were discarded from the cultures and fed with fresh medium in order to remove the false positive results by unabsorbed viral particles. Next, the supernatant of test wells was collected after 6 hours post-infection (hpi) every 6 hours and total RNA isolated using TRIzol (Invitrogen, *Cat No*: 15596018). Then, after reverse transcription of the extracted RNA, qRT-PCR was performed using SYBR-Green kit (Amplicon, Denmark, *Cat No:* A325402) according to manufacturer’s protocol in a StepOne plus real-time PCR system (Applied Biosystem). Specific primers to detect transcripts of ReoT3D were designed for L3 gene as follows: Reo-forward 5′-CGCCTCCCTTAAAGGTAAC-3′ (135 bp); Reo-reverse 5′-GACGTCTTAGTGATATGAAC-3′ (135 bp); and GAPDH internal control 5′- AGGAGTATGAC GAGTCCGGCCCCTC -3′. The median threshold cycle (_Ct_) value of the reference gene GAPDH was utilized to normalize the data and the relative quantification (ΔΔCt) method was used for data analysis by applying REST software (version 2009 2.0).

### GBM cell line treatment with ReoT3D

Three experimental groups including AD-MSCs, AD-MSCs loaded with ReoT3D, and ReoT3D were designed and then their potential in vitro anti-cancer effects against human glioblastoma cell line were assessed. First, AD-MSCs at passage 3 were infected with the nontoxic concentration of ReoT3D (MOI: 1 pfu/cells), incubated for 24 hours and then used for further experiments. Also, in the AD-MSCs group, AD-MSCs at passage 3 were applied for anti-cancer goals. To do anti-cancer experiments, U251 cells were cultured, detached, rinsed and then plated in the six-wells (3 × 10^5^ cells/well) and incubated at humidified chamber. Next, U-251 cultures at >70% confluency were treated with AD-MSCs, ReoT3D loaded AD-MSCs (at ratio 3:1 of U251 cells and AD-MSCs) and ReoT3D (at MOI: 1 pfu/cells of virus) incubated for 48 hours. Anti-cancer experiments were applied in in triplicate and mean of three values used for data analysis.

### Cell death assay

To determine the cell death rate in all test samples by flow cytometry assay, test cultures were collected, at 48 hpi, rinsed twice in PBS, resuspended at concentration of one million cells in PBS (500 μL), and then incubated in a 1x binding buffer comprised of AnnexinV-FITC (BioLegend, *Cat No*: *640945)* for 15 minutes at room temperature as manufacturer’s protocol.^26,27^ Finally, test cells were stained with propidium iodide (PI) (10 μL) at concentration of 30 μg/mL, the DNA content data were collected from a flow cytometry machine (FACSCalibur, BD Biosciences, San Jose) and analyzed through FlowJo version 7.6 software.

Also, qRT-PCR was conducted to measure the apoptotic and anti-apoptotic genes variation as described in previous sections. The gene-specific primers were listed in Table 1.

**Table 1:** The details of primers sequences that were used in real time PCR assay

**Primers List**	**Sequence ( 5’→3’)**	**Target gene**
h-P53-F	CAAAGACAGTAGGTTTATGAGG	Apoptotic
h- P53-R	CTTCTGAGGTCACCATTAG	
h-P21-F	CCATCTTTAGACAGTACGACC	
h- P21-R	GGAATCAGAGTCAAACACACAGA	
h-Bid-F	CCGACCACAAGGACTCG	
h-Bid-R	CACTACAGAAAGTGTGTTGTCA	
h-Bax-F	GACCTACTTTGGGACTTCGT	
h-Bax-R	GTCTCCTACTAACGGCGG	
h-Caspase8-F	AGGTCGTCCAAGTACAGTAGTAGG	
h-Caspase8-R	TTCTCAGACACGGGTTTAGTTGTT	
h-Caspase3-F	CTTATTATTGGTCCACGACAC	
h-Caspase3-R	ACTTTGTCATACGGCTGTT	
h-Bcl2-F	GTAGTTAGAAGTCGTGAGAGG	Anti-apoptotic
h-Bcl2-R	GTTGTAGTGTCTCCTTCATCTG	
h- Bcl-xL-F	CATAGGGTCGGCGGCAAG	
h- Bcl-xL-R	GGTGTAGGAGGCAGGTCG	
h- GAPDH-F	CTCATTTGGACTTAGAAACCTCAT	Internal control
h - GAPDH-R	GGACTTAACGATACACAGACC	

h: human.

### Cell cycle arrest distribution

To determine the cell cycle check-points distribution using flow cytometry, at 48 hpi, cultures were detached by trypsinization, rinsed in PBS, and then resuspended at concentration of one million cells as described before. Then, cells were resuspended in 450 μl ethanol, incubated for 24 h at 4 °C, and washed in cold PBS to be fixed. Fixed test cells were stained with 200 μl PI staining solution (eBioscience, *Cat No*: 00-6990-50) containing 50 μg/mL PI, 20 mg/mL of ribonuclease A, and 0.5% BSA for 30 minutes. The stained cells were washed in cold PBS and cell cycle distribution data collected from a flow cytometry machine (FACSCalibur, BD Biosciences, San Jose) and analyzed using FlowJo 7.6 software.

### Colony-forming ability

To determine the colony-forming ability of treated and non-treated cells, U251 cells at desired plating concentration (1×10^3^ cell for each Petri) were seeded in the 10 cm Petri dishes and incubated for 4 hours.^28^ After incubation, test cells were treated with AD-MSCs, ReoT3D, and ReoT3D loaded AD-MSCs for 10 days at humidified chamber. The survived colonies were washed with PBS, fixed with 4% paraformaldehyde and then incubated in 1% Giemsa staining solution (Sigma, USA, *Cat No:* 51811-82-6) at room atmosphere for colony staining. Finally, average size, number of colonies and colony formation area at colony forming pictures were calculated by image J software.

### Invasion assay

Migration rate of treated and non-treated cells was assessed by scratch assay. First, U251 cells (1×10^3^ cells/well) were seeded in the six-well plate, the cells equally were spread in the wells by gently moving from side to side and were incubated in the humidified chamber overnight. All of the test cultures at >80% confluency were scratched using a pipette tip, subsequently, media discarded and test wells replaced with fresh media comprised AD-MSCs, ReoT3D loaded AD-MSCs, and ReoT3D in the treatment cells. ImageJ software was applied for analysis of light microscopic images that were taken at 0 to 48 hpi.

### Statistical analysis

The raw data of experiments were analyzed by FlowJo 7.6, Image J, and REST software. The intragroup differences were defined by paired *t* test and Fisher’s least was used to compare differences between two groups while three/more groups analyzed by one-way ANOVA using GraphPad Prism software (version 6.0). All data were expressed as mean ± standard deviation (SD) and the *P* values <0.05 were defined as statistically significant.

## Results and Discussion

### AD-MSCs characterization

The aim of our study was to investigate anti-cancer properties and potential vehicle usage of AD-MSCs for ReoT3D delivery. First, ReoT3D was propagated and titrated and then AD-MSCs were isolated and characterized to these mains. The CPE of ReoT3D on L929 cells started at 18 hpi that increased in time-manner and maximum CPE was seen at~48 hpi. Virus titration using TCID_50_ assay calculated 1×10^7^ TCID_50_/mL of ReoT3D as our stock titer.

**Figure 1:**
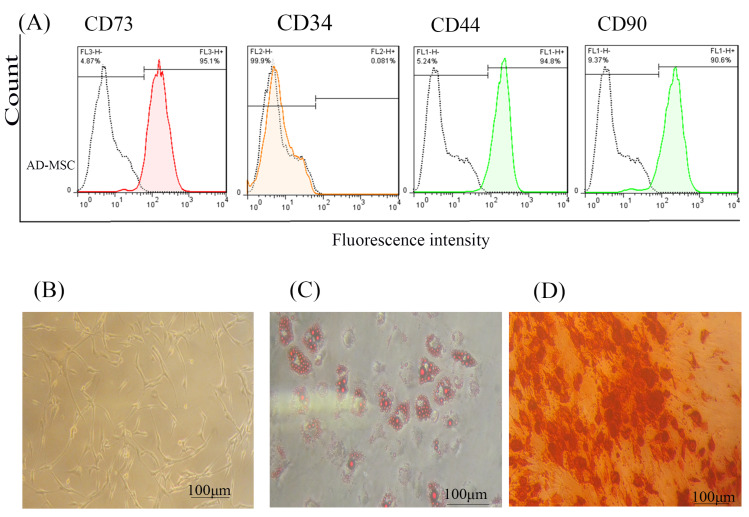
AD-MSCs characterization; **(A)** Flow cytometry assay to assess the CD markers in the surface of extracted AD-MSCs. Target cells were positive for CD73 (95.1%), and CD90 (90.6%) surface markers and were negative for CD44 and CD34 markers. **(B)** Morphology of AD-MSCs at passage 3. **(C)** Oil red staining to prove the adipogenic potential differentiation of AD-MSCs. **(D)** Alizarin-red staining to confirm the osteogenic potential differentiation of AD-MSCs. Scale bar = 100 μm. Abbreviations: AD-MSCs: adipose-derived mesenchymal stem cells.

In this study, AD-MSCs cells proved by flow cytometry, Alizarin-red, and Oil red staining according to previous studies.^29,30^ As shown in Figure 1A, our flow cytometry results demonstrated that isolated AD-MSCs expressed high levels of CD73 (95.1%) and CD90 (90.6%), and low levels of CD44 and CD34 markers. Fortunately, AD- MSCs were seen in the spindle and fibroblast-like shape (Figure 1B); this morphological shape was exhibited throughout subsequent subcultures. Also, Oil red staining proved AD-MSCs differentiation into adipocyte cells, which were incubated on 8 days (Figure 1C). Likewise, Alizarin-red staining confirmed that AD-MSCs could differentiate into osteogenic cells through incubation for 21 days (Figure 1D).

### Toxicity of ReoT3D on AD-MSCs and virus production ability by AD- MSCs

**Figure 2:**
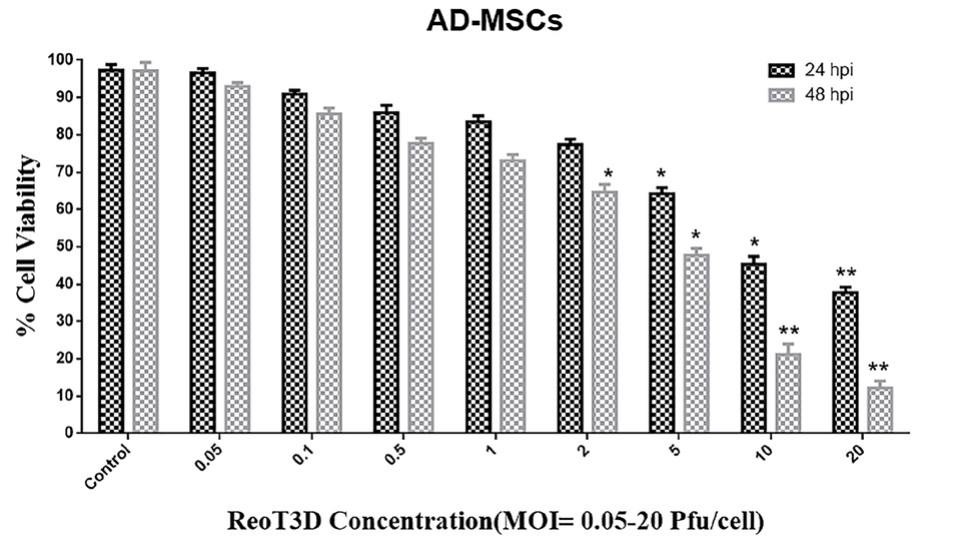
Cytotoxicity assay. Toxicity effect of ReoT3D on AD-MSCs cells was measured by MTT assay. AD-MSCs cells at passage 3 were infected with MOI 0.05- 20 pfu/cell of ReoT3D for 24, and 48 h. Toxicity was performed in triplicate for each concentration of RoeT3D and each value represents a mean ± SD (n = 3). Statistical significant levels are *P < 00.05 and ** P <0.01. Abbreviations: AD-MSCs: adipose-derived mesenchymal stem cells, MOI: multiplicity of infection.

To evaluate the viability of AD-MSCs against reovirus, these cells were infected with ReoT3D at MOI of 0.05- 20 pfu/cells for 24 and 48 hours. Viability results indicated that ReoT3D infection inhibited the proliferation of AD-MSCs in time and dose-dependent manner in comparison with control (Figure 2). Maximum and minimum toxicity of ReoT3D was observed at 24 hpi with MOI: 0.05 pfu/cells and 48 hpi with MOI: 20 pfu/cells, respectively. In the beginning, AD-MSCs were resistant to ReoT3D infection at MOI <2 pfu/cells, which significantly toxicity effect did not observe at these doses. Eventually, we found that AD-MSCs were susceptible for virus penetration; MOI <1 pfu/cells of ReoT3D were nontoxic for them. It has been shown that different cell lines show different behaviors and susceptibility to ReoT3D infection and some mouse and human cell lines are susceptible to reovirus infection.^31^ This variability among different infected cells can be related to source of cell, stages of the cell cycle, and genetic heterogeneity in virus population. Consistent with our results, MOI:1 pfu/cells of ReoT3D has been reported as optimum concentration for AD-MSCs loading.^32^

Despite the promising anti-cancer activity of OVs against the wide range of cancer cells with activated KRAS pathway, virus delivery and distribution at the tumor environment limited their therapeutic potential. To address these limitations, cell-based carriers were suggested and tested for OVs chaperoning to tumor sites.^33^ Moreover, recent studies confirmed the tumor-homing ability of stem cells after loading with OVs^15,34,35^; their compelling results increased the hops to effectively delivery of virus and maximizing anti-cancer action of OVs in future. Because of the intrinsic characteristic of MSCs to migrate toward tumor site, they protect the OVs against the host immune responses without any adverse effect on their biological activities.^36-38^ Furthermore, our data suggest that AD-MSCs are good hosts for the nontoxic concentration of ReoT3D and permissive producers for virus progenies. All of these promising characteristics demonstrate the potential of MSCs as a vehicle for OVs delivery.

### Apoptotic cells number in MSCs loaded with ReoT3D-treated cells was more than cultures treated with ReoT3D and MSCs

**Figure 4:**
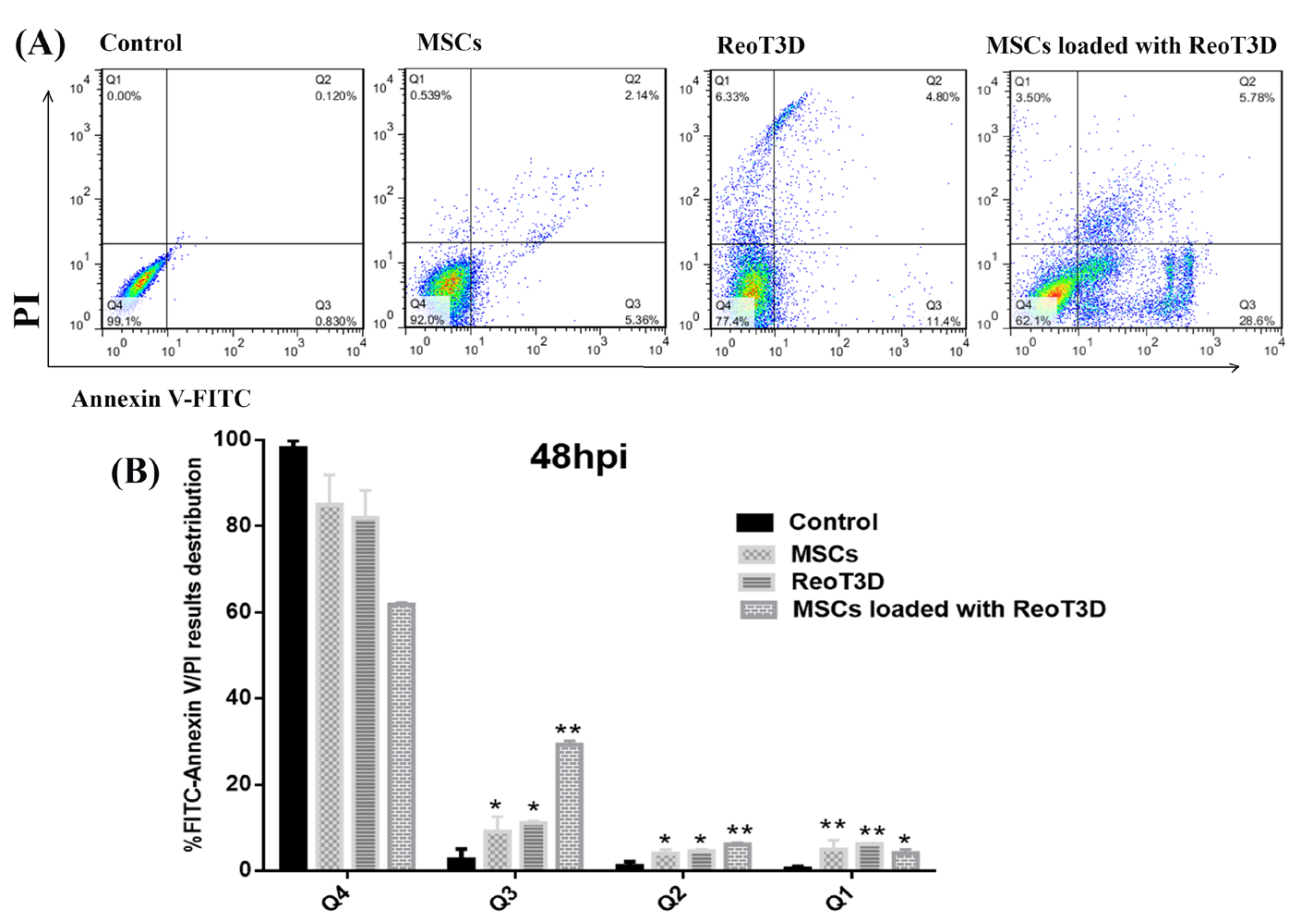
Apoptosis assays. Flow cytometry assay was performed to measure the rate of apoptotic cells in the treated cultures and compared with control at U-251 cells at 48 hpi. **(A).** Dot plots from flow cytometry divided stained cells into four sections: lower left quadrant (Q4) viable cells; bottom right quadrant (Q3) early apoptotic cells; top right quadrant (Q2) late apoptotic cells/cells without membrane integrity, and top left quadrant (Q1) necrotic cells**. (B).** Bar graph (%) of stained cell distribution is shown microscope, * P <0.05, and ** P < 0.01 (n=3). Abbreviations: MSCs: mesenchymal stem cells, MOI: multiplicity of infection, Q: quadrant.

Previous reports demonstrated that ReoT3D induces apoptosis and suppresses growth of *KRAS* activated cancer cells.^12^ This genetic defect was observed >80% of GMB cells.^39^ Therefore, effective targeting of this pathway by ReoT3D might be considered as an appropriate method for treatment of GMB.^40^ It has been shown that OVs, especially oncolytic reovirus, are capable of targeting cancer stem cells and triggering apoptosis in the cells^41,42^ Also, MSCs could induce apoptosis in the cancer cells.^43^ Here, the death rate in all treated and non-treated cells was examined by flow cytometry at 48 hpi. Generally, flow cytometry results indicated that cell death rate in all treated cultures significantly induced in comparison with control (Figure 4A). Our Annexin V-FITC experiments demonstrated that AD-MSCs, and ReoT3D distinctly induced the apoptosis in the cells with maximum impact by ReoT3D loaded AD-MSCs (Figure 4B), suggesting the potential of AD-MSCs in promoting the impact of ReoT3D in induction of apoptosis. Altogether, our results suggest that synergistic anti-cancer impact of ReoT3D and AD-MSCs may be due to the targeting of normal cancer cells and cancer stem cells.

### ReoT3D loaded MSCs and ReoT3D enhance expression of apoptotic genes and reduce anti-apoptotic genes

**Figure 5:**
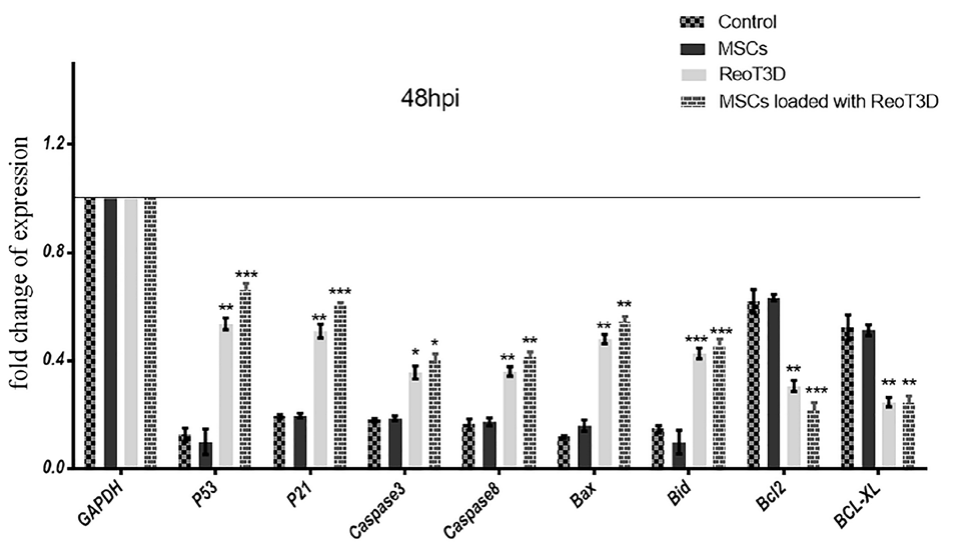
Real-time PCR assay. Graphic illustration of apoptotic and anti-apoptotic genes expression level in the test samples that was measured by real-time PCR at 48 hpi. The results are representatives of three independent runs, * P <0.05, ** P <0.01, and *** P <0.001 (n=3). Abbreviations: MSCs: mesenchymal stem cells, hpi: hours post-infection.

Also, to confirm the effect of treatments on apoptosis, the relative expression levels of death receptor-(exogenous) and mitochondrial-caspase-dependent (endogenous) apoptotic and anti-apoptotic genes were analyzed by qRT-PCR at 48 hpi. The results revealed a significant induction in P53, P21, Caspase3, Caspase8, Bax, and Bid genes expressions in the cells when treated with ReoT3D loaded AD-MSCs. However, anti-apoptotic genes expressions including Bcl-2 and Bcl-xL significantly decreased by this treatment. Furthermore, both exogenous and endogenous apoptosis genes expression level in ReoT3D treated sample was somewhat similar in AD-MSCs loaded with ReoT3D culture but a remarkable difference were not seen between the expression of Bcl-2 and Bcl-xL at ReoT3D treated and control cells. At the front, real time PCR did not show significant difference between expression of exogenous and endogenous apoptosis and also anti-apoptosis markers in AD-MSCs treated cells (Figure 5).

**Figure 3:**
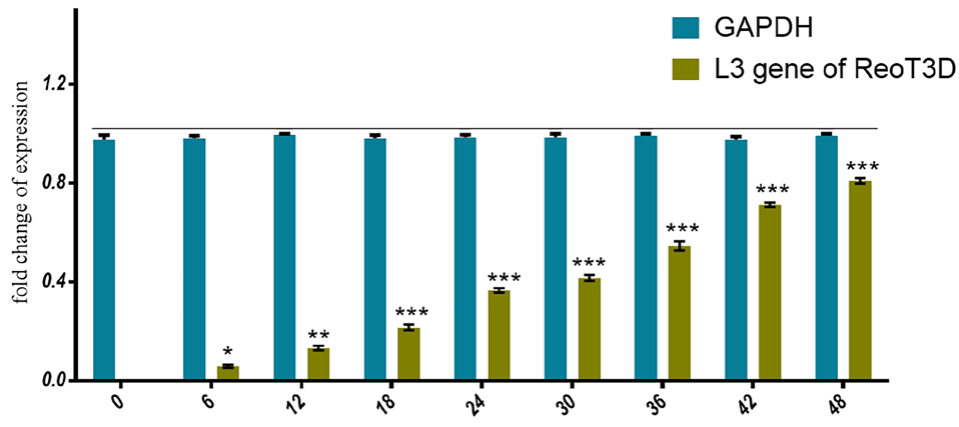
ReoT3D production in AD-MSCs. Real-time PCR used to assess the capability of AD-MSCs to produce ReoT3D. AD-MSCs were infected with virus and the viral genome level in the supernatant of cultures was measured after 6 hpi every 6 h. Results are presented as mean ± SD (n=3), *P < 0.05, **P < 0.01 and ***P < 0.001. Abbreviations: AD-MSCs: adipose-derived mesenchymal stem cells.

Moreover, after measuring the nontoxic concentration of ReoT3D on AD-MSCs, the capability of these cells to virus production was assessed by real-time PCR assay. Our results from the evaluation of the viral genome in the supernatant of infected culture revealed that the genomes were slightly detectable at early hours upon infection (about 6 hpi), which increased by the time and reached the maximum level at 48 hpi (Figure 3).

It has been shown that ReoT3D performed its oncolysis activity through endogenous pathway.^44^ Furthermore, the apoptosis induction by ReoT3D in both exogenous and endogenous pathways has been reported^45-47^; our results were consistent with their findings. In addition, our gene expression results revealed that ReoT3D loaded AD-MSCs distinctly induced TRAIL apoptosis genes expression more than caspase-dependent apoptosis pathway. The MSCs loaded with OVs increased their anti-cancer efficiency by TRAIL pathway.^48,49^

### ReoT3D loaded MSCs with result in G1 and G2 fraction induction with S phase reduction in U-251 cells

**Figure 6:**
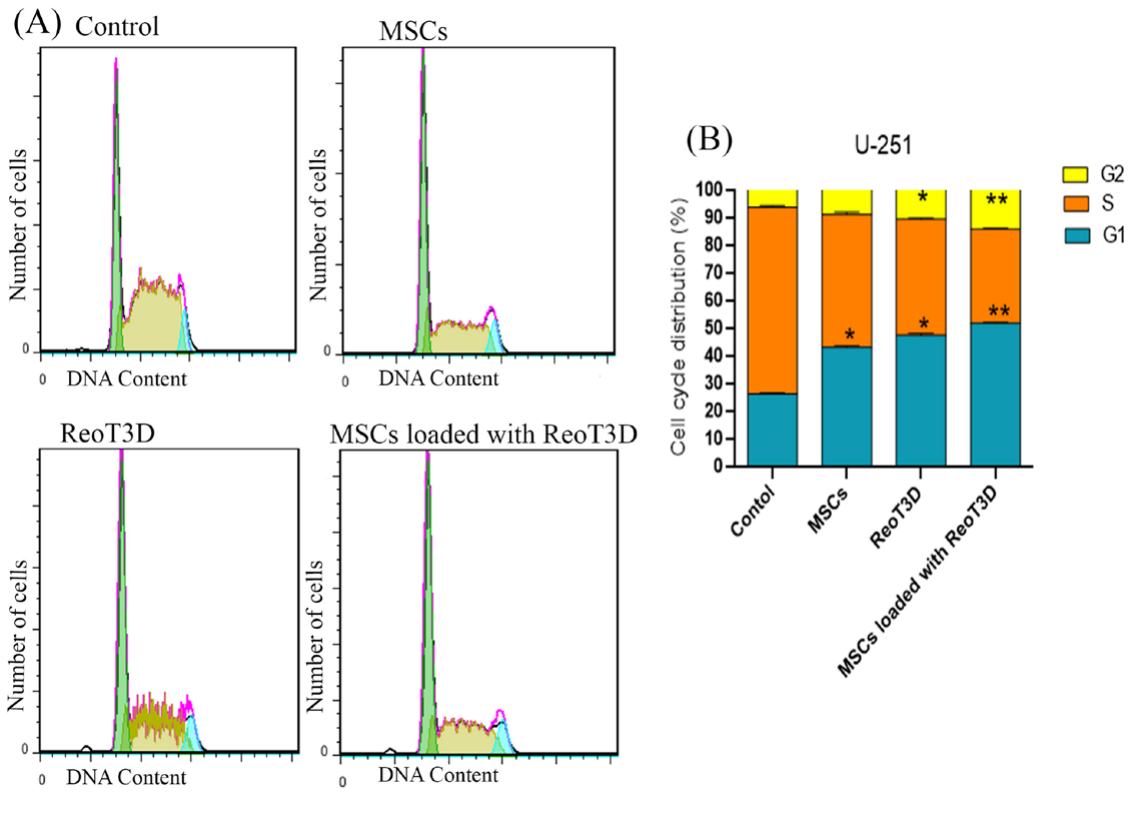
Cell cycle distribution. Flow cytometry was performed to analyze the differences in cell cycle distribution at test cultures in comparison with control at 48 hpi. **(A)** Representative plots of one set of triplicate experiments are shown. Cells were sorted in G1, S, and G2 phases of the cell cycle based on DNA contents after staining with PI. **(B)** Bar graph (%) of target cells distribution in each phase of cell cycle, * P <0.05, and **P < 0.01 (n=3). Abbreviations: MSCs: mesenchymal stem cells, hpi: hours post-infection.

ReoT3D induced apoptosis and cell cycle arrest at G1/S and G2/M in infected cells.^50,51^ Generally, our results from cell cycle analysis revealed that all treatment groups significantly arrested growth of treated cells. Cell cycle distribution at the AD-MSCs- treated cells showed only increase of cell number at G1 phase and also ReoT3D were effective to suppress the growth of U-251 cells in G0/G1 and G2M phases (Figure 6A-B). Furthermore, maximum arrest at G0/G1 and G2M check points with marked S-phase ablation were seen in AD-MSCs loaded with ReoT3D cultures. Altogether, the results suggest that AD-MSCs enhance the oncolysis ability of ReoT3D.

### ReoT3D loaded MSCs reduced the colony-forming ability and migration rate of U-251 cells 

**Figure 7:**
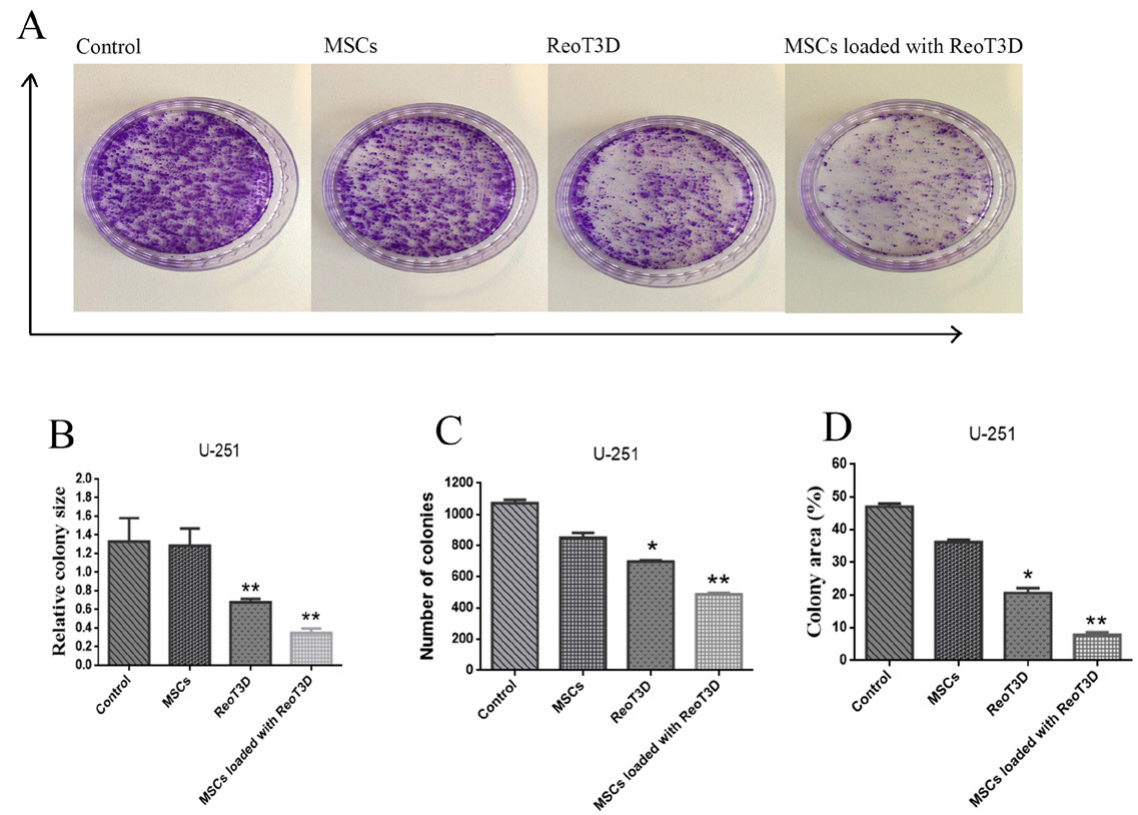
Colony forming assay. **(A)** U-251 cells colony viability in all test samples was measured after seeding 1×10^3^ cells per each 10 cm petri dish. **(B)** Graphic illustration of relative colony size.** (C)** Graphic illustration of colony numbers. **(D)** Graphic illustration of the colony area. The results are representatives of three independent runs and obtained images were analyzed by ImageJ software, * P <0.05, and **P< 0.01 (n=3). Abbreviations: MSCs: mesenchymal stem cells.

**Figure 8:**
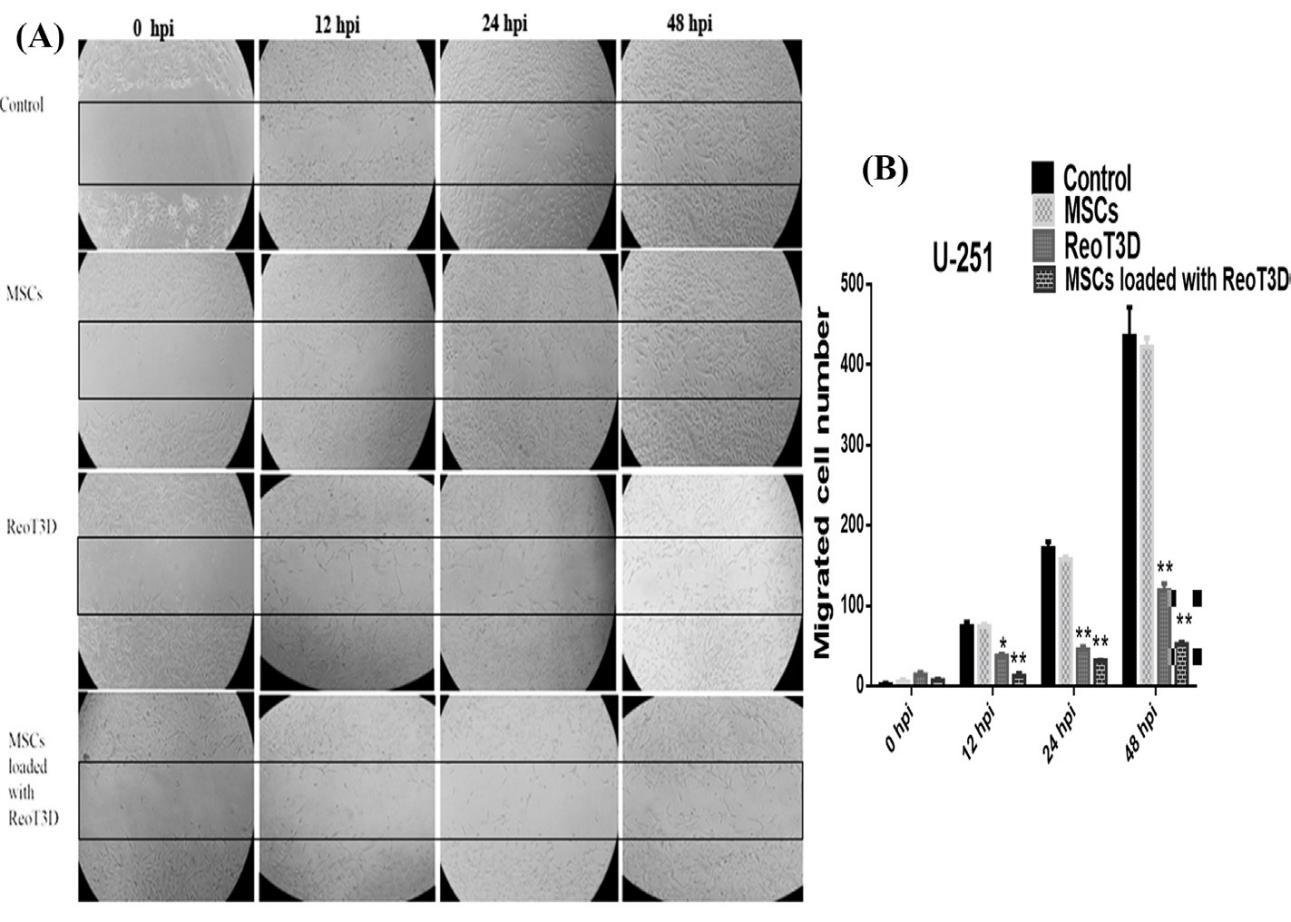
Scratch assay**. (A)** U-251 cells migration in all test samples was measured by scratch assay at 0, 12, 24, and 48 hpi. **(B)** Graphic illustration of migrated cell numbers at all treatments. The results are representatives of three independent runs, and results are given as migrated cell numbers, * P <0.05, ** P <0.01 (n=3). Abbreviations: MSCs: mesenchymal stem cells, hpi: hours post-infection.

Colony-forming analysis revealed a maximum reduction in the relative colony size, the number of colonies and colony areas in the cells treated with RedoT3D loaded AD-MSCs. Furthermore, ReoT3D showed a desirable effect on the colony-forming ability of U-251 cells; however, AD-MSCs did not affect this ability (Figure 7A-D). Also, to assess the impact of ReoT3D loaded MSCs and ReoT3D on migration ability of the cells, we applied migration assay through the light microscopic at 0, 12, 24, and 48 hpi. Our results showed that MSCs loaded with ReoT3D and ReoT3D decreased the number of migrated cells (Figure 8A-B). Our data were consistent with previous studies which reported that MSCs enhanced oncolysis ability of OVs.^48,52^ Altogether, we demonstrate that AD-MSCs as host for OVs were capable of producing the viral progenies with delay and strongly increasing the anti-cancer impacts of oncolytic reovirus by induction of apoptosis and reduction of cell cycle, colony-forming ability and migration rate of GMB cell line. Previous studies reported that OVs loaded MSCs performed its tumoricidal impact by virus protection from neutralizing antibodies; OVs delivery to tumor sites by Trojan horse strategy, increase the anti-cancer potential of OVs.^15,53,54^ More investigations are needed to clarify the exact molecular mechanisms underlying the impact of MSCs on anti-cancer properties of RedoT3D.

## Conclusion

Our results highlighted that MOI >1 pfu/cell of ReoT3D was tolerable for characterized AD-MSCs. Furthermore, the results revealed that AD-MSCs increased the anti-cancer impacts of ReoT3D on the U-251 cells. Collectively, the current in vitro data support this idea that AD-MSCs could be considered as an effective vehicle delivery candidate for OVs.

## Ethical Issues

Not applicable.

## Conflict of Interest

The authors declare no conflicts of interest.

## Acknowledgments

We thank Aja University of Medical Sciences for financial supports [grant number: 97000676].
